# Identification of differentially expressed genes of blood leukocytes for Schizophrenia

**DOI:** 10.3389/fgene.2024.1398240

**Published:** 2024-06-26

**Authors:** Feifan Wang, Yao Fan, Yinghui Li, Yuan Zhou, Xin Wang, Mengya Zhu, Xuefei Chen, Yong Xue, Chong Shen

**Affiliations:** ^1^ Department of Epidemiology, School of Public Health, Nanjing Medical University, Nanjing, China; ^2^ Department of Clinical Epidemiology, Jiangsu Province Geriatric Institute, Geriatric Hospital of Nanjing Medical University, Nanjing, China; ^3^ Department of Medical Psychology, Huai’an Third Hospital, Huai’an, China; ^4^ Department of Medical Laboratory, Huai’an Third Hospital, Huai’an, China

**Keywords:** Schizophrenia, RNA sequencing, WGCNA, bioinformatics, gene expression

## Abstract

**Background:**

Schizophrenia (SCZ) is a severe neurodevelopmental disorder with brain dysfunction. This study aimed to use bioinformatic analysis to identify candidate blood biomarkers for SCZ.

**Methods:**

The study collected peripheral blood leukocyte samples of 9 SCZ patients and 20 healthy controls for RNA sequencing analysis. Bioinformatic analyses included differentially expressed genes (DEGs) analysis, pathway enrichment analysis, and weighted gene co-expression network analysis (WGCNA).

**Results:**

This study identified 1,205 statistically significant DEGs, of which 623 genes were upregulated and 582 genes were downregulated. Functional enrichment analysis showed that DEGs were mainly enriched in cell chemotaxis, cell surface, and serine peptidase activity, as well as involved in Natural killer cell-mediated cytotoxicity. WGCNA identified 16 gene co-expression modules, and five modules were significantly correlated with SCZ (*p* < 0.05). There were 106 upregulated genes and 90 downregulated genes in the five modules. The top ten genes sorted by the Degree algorithm were *RPS28*, *BRD4*, *FUS*, *PABPC1*, *PCBP1*, *PCBP2*, *RPL27A*, *RPS21*, *RAG1*, and *RPL27*. *RAG1* and the other nine genes belonged to the turquoise and pink module respectively. Pathway enrichment analysis indicated that these 10 genes were mainly involved in processes such as Ribosome, cytoplasmic translation, RNA binding, and protein binding.

**Conclusion:**

This study finds that the gene functions in key modules and related enrichment pathways may help to elucidate the molecular pathogenesis of SCZ, and the potential of key genes to become blood biomarkers for SCZ warrants further validation.

## Introduction

Schizophrenia (SCZ) is a severe mental disorder with brain dysfunction of unknown etiology, often with hallucinations, delusions, and functional deterioration (Owen et al., 2016; Marder et al., 2019; Jauhar et al., 2022). The results of the China Mental Health Survey (CHMS) released in 2019 show that the lifetime prevalence rate of SCZ in China is about 0.6%, and the disability rate is relatively high (Huang et al., 2019). According to the Global Disease Burden study, the disability lost life years (DALY) of SCZ ranks the 20th among all diseases and the 3rd among mental disorders in the world, and the 17th among all diseases and the 3rd among mental disorders in China (GBD, 2019 Mental Disorders Collaborators, 2022), respectively.

A complex interplay of genetic, biological, and environmental factors is associated with SCZ (Marder and Cannon, 2019). Psychiatric symptoms are related to the dysfunction of dopaminergic neurotransmission, and abnormal glutamate signaling relevant to negative symptoms and cognitive impairment (Moghaddam and Javitt, 2012; Howes and Murray, 2014), with obvious molecular pathological changes in the central nervous system (Jauhar et al., 2022). However, due to the difficulty in collecting brain tissues from deceased patients, research and application in the central nervous system are limited. In contrast, peripheral blood mononuclear cells (PBMCs) have become an important research carrier of SCZ, with a more ideal way to collect quickly and conveniently, and can reflect some molecular characteristic changes in the central nervous system (Herberth et al., 2011; Gardiner et al., 2013). After PBMCs pass through the blood-brain barrier and migrate and infiltrate the brain specifically, they can participate in pathological reactions (Hickey, 1999; Ransohoff et al., 2003; Cayrol et al., 2008). Leukocytes can infiltrate through the fenestrated endothelium of the choroid plexus matrix, migrate to the villi through the matrix core, interact with the epithelial cells of the choroid plexus, and enter the cerebrospinal fluid at its formation site (Carrithers et al., 2002). Leukocytes can also pass through the internal carotid artery, infiltrate through the capillaries on the surface of the brain, and then enter the subarachnoid space and the space around the Virchow-Robin vessels through small veins (Hickey, 1999; Kerfoot and Kubes, 2002).

Researchers have demonstrated that PBMCs exhibit gene expression patterns similar to those in the brain (Bowden et al., 2006; Sullivan et al., 2006). Sullivan et al. conducted the secondary data analysis on 17 microarray datasets (1 whole blood and 16 different brain regions) and found significant gene expression similarity between whole blood and multiple central nervous system tissues (prefrontal cortex, amygdala, whole brain) (Sullivan et al., 2006). The median non-parametric correlation between transcripts present in whole blood and the central nervous system was approximately 0.5. The comparability of gene expression profiles in cerebellar tissues and PBMCs from the same human participant was evaluated directly by Rollins et al. (2010). In the entire transcriptome, they observed a blood-brain Pearson correlation coefficient of 0.64 in a set of 17,859 probes. When only examining the probe set that met the minimum expression threshold, they observed a stronger correlation of gene expression between the brain and blood (*r* = 0.98). Peripheral blood alterations may, to some extent, mirror central nervous system changes.

In this study, we conducted RNA sequencing with peripheral blood leukocyte samples of SCZ cases and healthy controls to identify differentially expressed genes (DEGs), performed weighted gene co-expression network analysis (WGCNA), and identified candidate blood biomarkers for SCZ.

## Methods

### Study population

The characteristics of the RNA sequencing samples of 9 SCZ cases were recruited from the Third People’s Hospital of Huai’an City, Jiangsu Province, and 20 healthy controls recruited from the community survey were described in Supplementary Table S1. All patients with SCZ were in the acute episode and lacked systemic and effective antipsychotic drug treatment. The diagnostic criteria for the SCZ cases referred to the International Classification of Disease (ICD-10). All the cases and controls were free of neuroimmunological and neurodegenerative disorders, cardiovascular disease, cerebral vascular disease, serious infections, and surgery.

This study was done in full compliance with the Helsinki Declaration. The protocol and consent form were approved by the Institutional Review Board of Nanjing Medical University, China. All participants or their guardians provided signed informed consent.

### Blood sample collection and RNA sequencing

Over 8 h of fasting of patients with SCZ at admitted, venous blood was collected using an anticoagulant tube with ethylene diamine tetraacetic acid (EDTA)-k2. White blood cells were rapidly separated and treated with human peripheral blood RNA preservation solution (Eaglink, EGEN 2026, China). Total RNA was extracted using the magnetic bead method (Yuan, Yu-BR02-1, China). NanoPhotometer Spectrophotometer (IMPLEN, CA, United States) was used for detecting sample purity. Qubit 3.0 Fluorometer (Life Technologies, CA, United States) was used to detect total RNA concentration. The total RNA library was qualified, and RNAs were sequenced by the platform of Illumina, Novaseq6000.

### Analysis of differentially expressed genes (DEGs)

The quality control process for sequencing data encompasses the following steps: removing adapter sequences; discarding bases at the beginning and end of reads with quality values below 20; shifting the window from the 5′ end to eliminate bases with an average quality value below 20; and filtering reads with lengths below 50 bp. Sequencing data was normalized using fragments per kilobase of exon model per million mapped fragments (FPKM). The R package *DESeq2* (v1.38.3) in R software (v4.2.3) was used to screen DEGs. DEGs were identified with *p-*value < 0.05 and |log_2_FC| > 1. A volcanic map was drawn to display the overall distribution of DEGs in two aspects: fold change and significance levels. A hierarchical clustering heatmap was created to cluster DEGs with the same or similar expression behavior. Volcano and heat maps were both plotted using the R package *ggplot2* (v3.4.1).

### Weighted gene co-expression network analysis (WGCNA)

Based on RNA sequencing data, weighted gene co-expression network analysis was performed using the R package *WGCNA* (v1.72). A co-expression correlation matrix was constructed by calculating the Pearson correlation coefficient between genes and then transformed into a weighted adjacency matrix using a power adjacency function. The definition of weighting was to take the correlation coefficient between genes to the Nth power (N was the soft threshold). To ensure that the connections between genes in the constructed network followed a scale-free network distribution with high independence (*R* ^2^ > 0.80), the optimal soft threshold in this study was set to 14 (*R* ^2^ = 0.85). We then created a topological overlap matrix (TOM) by converting the adjacency matrix. Hierarchical clustering analysis was performed according to the TOM. The clustering results were segmented based on predetermined criteria (minimum module size = 30, cut height = 0.25) to produce distinct gene modules, which were represented by the branches and various hues of the clustering tree. The study also explored the correlation between gene modules and disease status, and based on various correlation results, the modules most relevant to the disease status were identified (|correlation coefficient| > 0.5 and *p-*value < 0.05). This study first calculated the correlation matrix between modules and genes, by the correlation between gene expression within the module and module eigengenes (MEs), for module membership (MM). Then the correlation matrix between traits and genes was calculated, by the Pearson correlation coefficient between gene expression within the module and disease status, for gene significance (GS). Finally, we combined the two correlation matrices and specified the significant modules for analysis. The criteria for screening key genes within the modules were |MM| > 0.8 and |GS| > 0.2.

### Functional enrichment analysis

Gene ontology (GO) and Kyoto encyclopedia of genes and genomes (KEGG) enrichment analysis were performed with R package *clusterProfiler* (v4.6.2), to obtain the biological functions and enrichment pathways of the DEGs. The criterion for identifying linked KEGG pathways and GO functions was set at *p* < 0.05.

## Results

### Differentially expressed genes analysis

**FIGURE 1:**
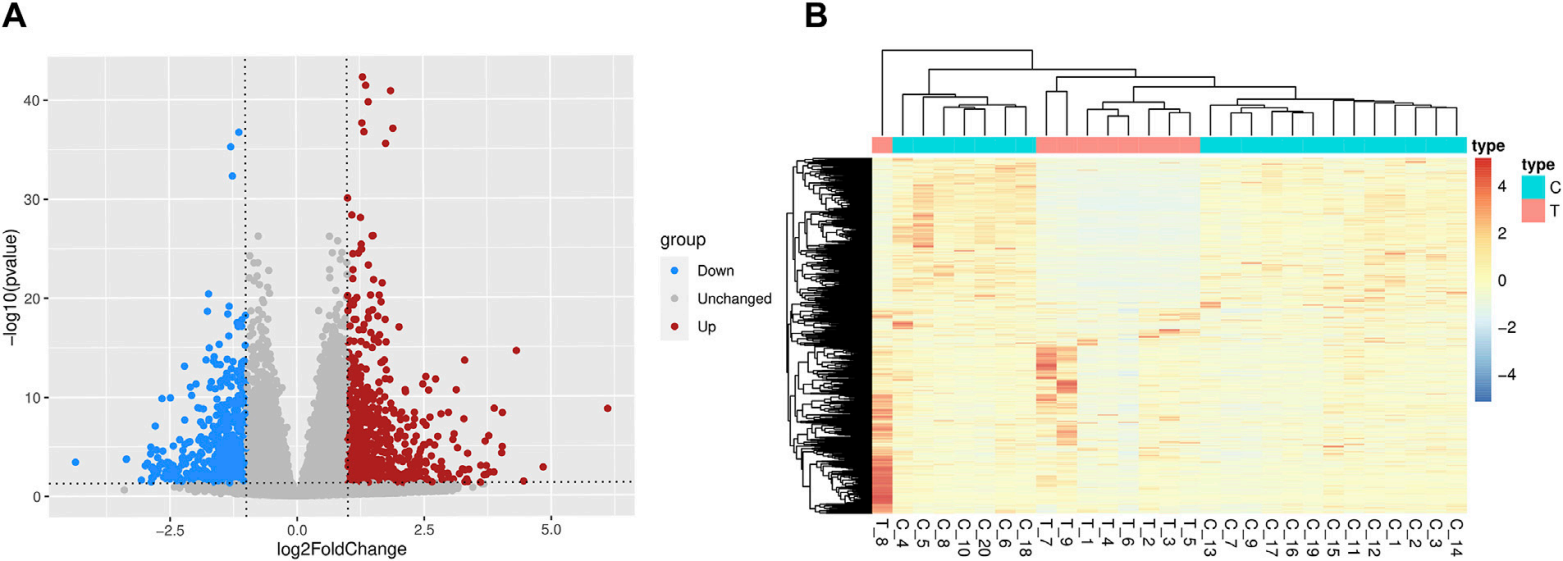
Differentially expressed genes between SCZ and health control blood samples. **(A)** The volcano plots. Red denotes the upregulated genes and blue denotes the downregulated genes. **(B)** The heatmaps of differentially expressed genes. T = SCZ cases, C = health controls.

Supplementary Figure S1 displayed a flowchart outlining the general study analysis. 1,205 DEGs were screened (*p-*value < 0.05 and |log_2_FC| > 1), of which 623 genes were upregulated and 582 genes were downregulated (Figure 1). There are some DEGs in this study that were reported to be significant in the previous studies and relevant databases (Supplementary Table S6; Supplementary Figures S2, S3).

### Biological and functional enrichment analysis

The GO enrichment analysis results (Supplementary Table S2) showed that DEGs were mainly enriched in cell chemotaxis (biological process, BP), cell surface (cellular component, CC), and mRNA 3′-UTR AU-rich region binding (molecular function, MF). The results of KEGG pathway enrichment analysis showed that DEGs mainly participated in Parkinson’s disease, Oxidative phosphorylation, Natural killer cell-mediated cytotoxicity, Metabolism of xenobiotics by cytochrome P450, Taste transformation, Graft *versus* host disease, African trypanosomiasis, Osteoclast differentiation, Prostate cancer, Nitrogen metabolism.

### Analysis of co-expression modules

**FIGURE 2:**
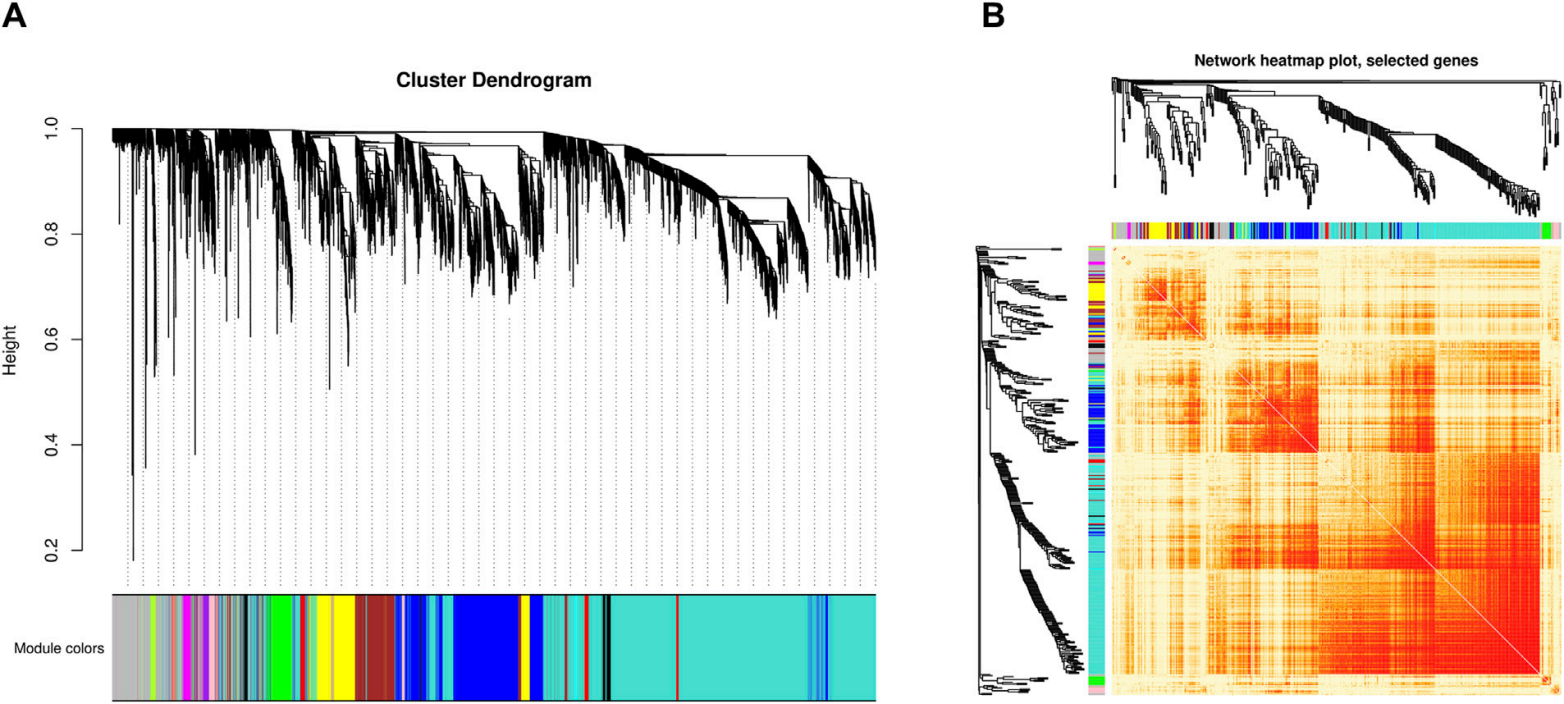
Identification of co-expressed modules by WGCNA. **(A)** Cluster dendrogram of all genes. The x-axis represents the gene modules, the y-axis represents the network distance with values closer to zero indicating higher gene expression similarity. **(B)** Heatmap plot of 400 selected co-expressed genes. Each row and column corresponds to a gene, light color denotes low topological overlap, and progressively darker red denotes higher topological overlap. Darker squares along the diagonal correspond to modules.

The weighted gene co-expression network analysis identified 16 gene co-expression modules (Figure 2A). The genes in the grey module cannot be clustered into any other modules and did not have co-expression relationships (Supplementary Table S3).

**FIGURE 3:**
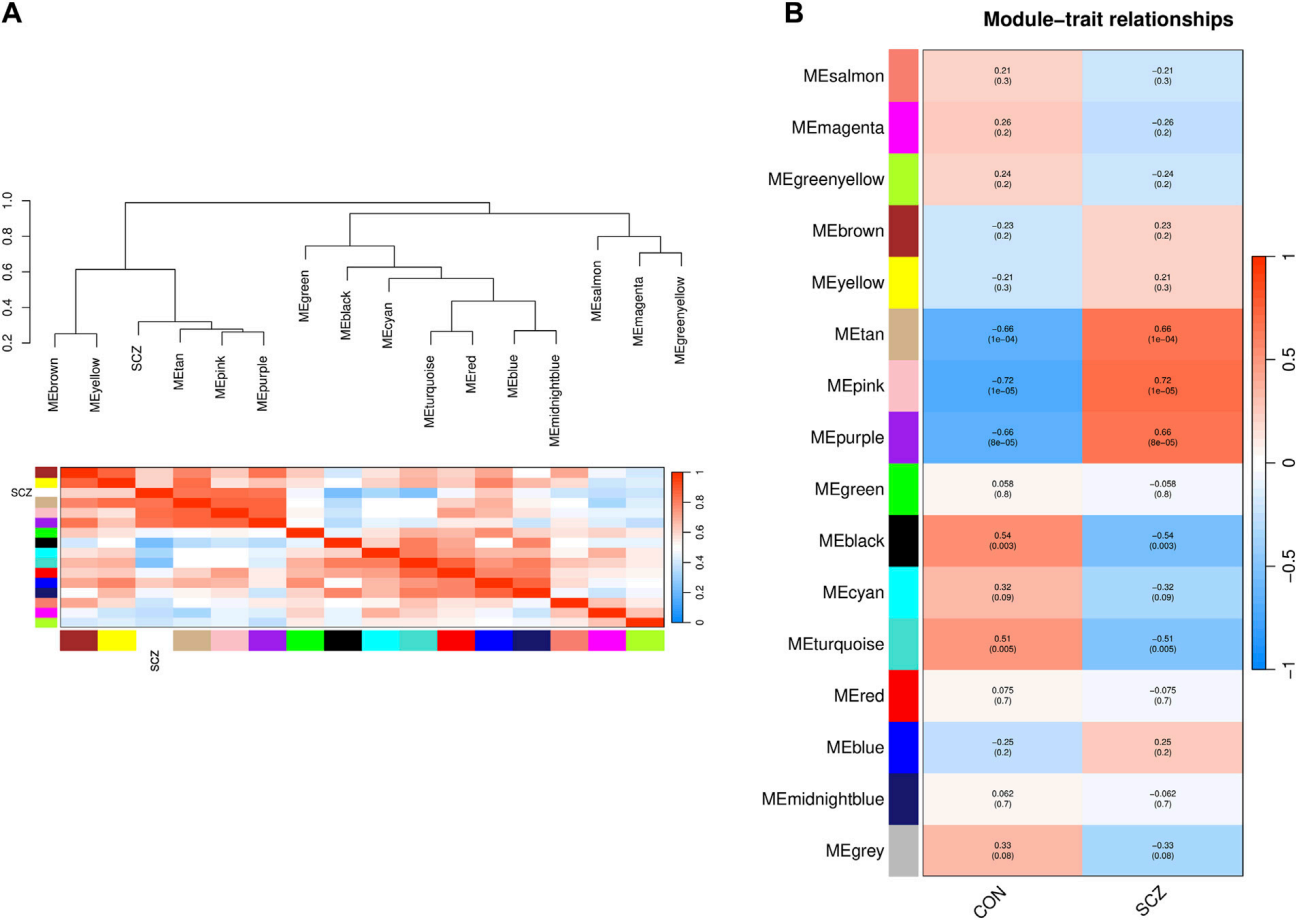
Identification of the relationship of modules and disease status by WGCNA. **(A)** Dendrogram of Module Eigengene (ME) and heatmap of the adjacencies of modules. Each row and column in the heatmap corresponds to one module eigengene (labeled by color) or SCZ. In the heatmap, blue represents low adjacency (negative correlation), while red represents high adjacency (positive correlation). **(B)** Correlation of modules and disease status. Red represents a positive correlation with disease status, and blue represents a negative correlation with disease status.

The characteristic genes of each module were plotted as a correlation heatmap between co-expression modules and the disease (Figure 3A). There were five modules significantly associated with SCZ (|correlation coefficient| > 0.5 and *p*-value < 0.05). Among them, the genes in the tan, pink, and purple modules were generally positively correlated with SCZ, indicating that the genes in these modules were mostly overexpressed in SCZ patients (Figure 3B). The genes in the black and turquoise modules were generally negatively correlated with SCZ, indicating that they were mostly low expressed in SCZ patients (Figure 3B).

### Gene expression patterns and functional enrichment analysis of related modules

**FIGURE 4:**
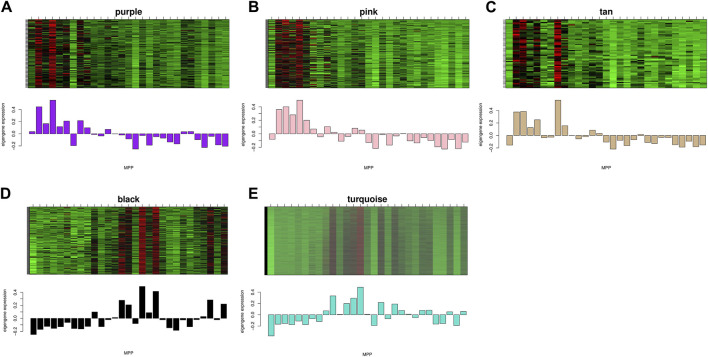
Expression levels of all genes in the 5 key modules and the corresponding ME expression values of key modules versus the same sequenced samples. Red represents upregulated genes and green represents downregulated genes.

**FIGURE 5:**
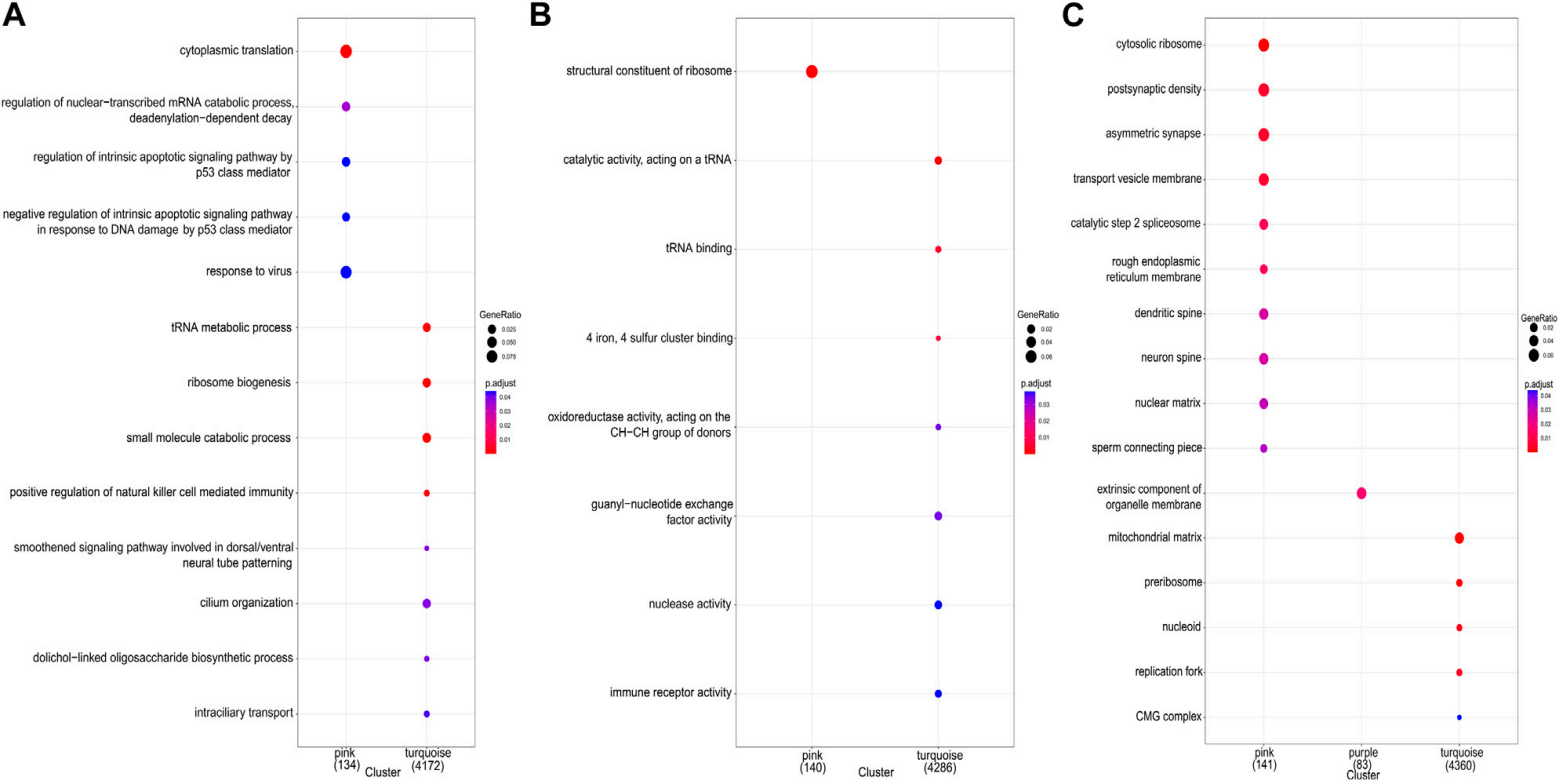
GO enrichment analysis for all genes in key modules. **(A)** GO terms of biological process (BP). **(B)** GO terms of molecular function (MF). **(C)** GO terms of cellular component (CC).

The study demonstrated the gene expression patterns of five modules through heat maps and changes in modules’ eigenvalues across all samples and conducted functional enrichment analysis on module genes (Figure 4). The pink module genes were mainly enriched in CC, such as cytosol ribosomes, postsynaptic density, and asymmetric synapses. The turquoise module genes were mainly involved in BP, such as tRNA metabolic process, ribosome biogenesis, and small molecule catabolic process. The purple module genes were mainly enriched in the extrinsic component of organelle module (Figure 5).

### Identify key genes of related modules and functional enrichment analysis

**FIGURE 6:**
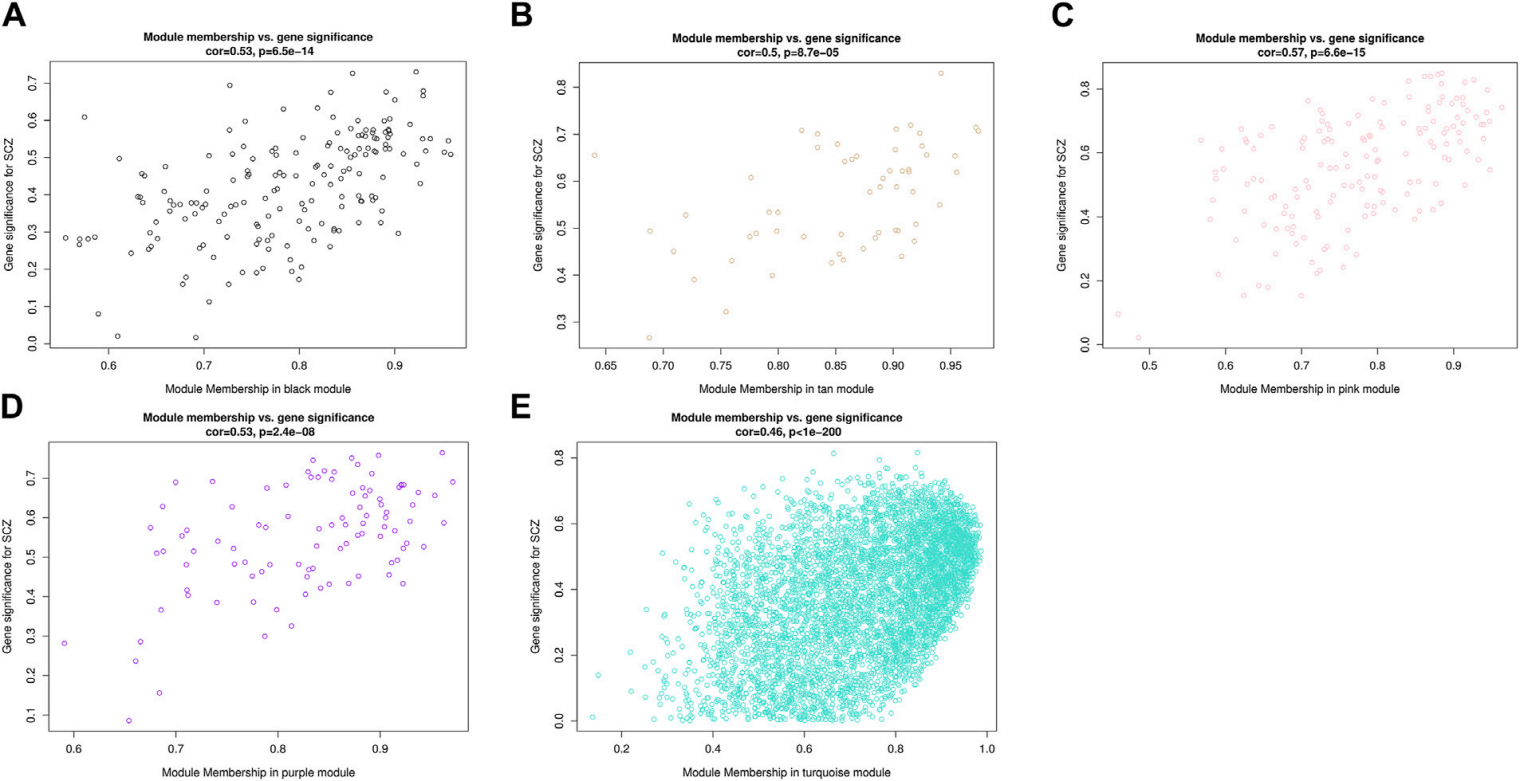
Scatterplots of gene significance (GS) for disease status versus module membership (MM) in five modules. The x-axis represents the correlation between gene expression within the module and MEs, the y-axis represents the Pearson correlation coefficient between gene expression within the module and disease status.

Combining two correlation matrices to generate scatter plots allowed us to screen for genes of interest. Each point in the scatter plot represented a gene, with the abscissa value representing the correlation between genes and modules, and the ordinate value representing the correlation between genes and phenotypic traits. The results showed that the correlation between MM and GS in all five modules was statistically significant (*p* < 0.001), indicating that genes highly correlated with SCZ had a stronger correlation with their corresponding modules (Figure 6). Among them, the pink module had the highest correlation between MM and GS (correlation coefficient = 0.57, *p* = 6.6e–15). Using the conditions of |MM| > 0.8 and |GS| > 0.2 to screen key genes, we identified a total of 196 key genes in five modules, including 106 upregulated genes and 90 downregulated genes (Supplementary Table S4). Utilizing Cytoscape to determine the degree of key genes, the top ten genes were *RPS28*, *BRD4*, *FUS*, *PABPC1*, *PCBP1*, *PCBP2*, *RPL27A*, *RPS21*, *RAG1* and *RPL27*. *RAG1* and the other nine genes belonged to the turquoise and pink module respectively. These 10 genes were involved in processes such as Ribosome, Ferroptosis, cytoplasmic translation, translation, viral RNA genome replication, ribonucleoprotein complex, RNA binding, protein binding, and structural constituent of ribosome.

## Discussion

In this study, by using the RNA sequencing data of peripheral leukocytes, we conducted GO enrichment analysis, KEGG pathway enrichment analysis, and weighted gene co-expression network analysis to screen for DEGs of SCZ, and successfully identified five gene modules associated with disease status, including 106 upregulated genes and 90 downregulated genes. The top ten genes sorted by the Degree algorithm were *RPS28*, *BRD4*, *FUS*, *PABPC1*, *PCBP1*, *PCBP2*, *RPL27A*, *RPS21*, *RAG1*, and *RPL27*.

Functional enrichment analysis showed that DEGs mainly participated in pathways such as Oxidative phosphorylation, Natural killer cell-mediated cytotoxicity, and Metabolism of xenobiotics by cytochrome P450. Previous studies have shown that the occurrence of SCZ is related to immunity and oxidative stress (McCutcheon et al., 2020). Stein et al. found a significant decrease in creatine kinase activity and oxidation-reduction ratio in the prefrontal cortex of SCZ patients, indicating that during the SCZ process, the energy generation mode gradually shifted from oxidative phosphorylation to glycolysis, which was related to oxidation-reduction imbalance and mitochondrial dysfunction (Stein et al., 2023). Meanwhile, well understanding of the relationship between pharmacogenetics, metabolic function and SCZ would help improve the therapeutic efficacy of second-generation antipsychotics (Płaza et al., 2022). Despite the fact that the enrichment pathways we found were associated with SCZ, the varying ages of our sequencing samples could possibly be the cause of these pathways.

At present, the etiology and mechanisms of SCZ are still unclear, but genetic factors are the most influential with strong evidence supported (Cannon et al., 1998; International Schizophrenia et al., 2009; Lam et al., 2019). For the key genes discovered, we found relevant literature evidence to support their possible involvement in the pathogenesis and development of SCZ. *BRD4* (bromodomain-containing protein 4) belongs to the BET family and is a class of epigenetic regulatory factors involved in a series of cellular functions (Wu and Chiang, 2007; Dey et al., 2009; Zhao et al., 2011). *BRD4* is widely distributed in the brain and typically exists in neurons. *BRD4* initiates gene transcription by recognizing acetylated histones, which are associated with many psychiatric disorders (Korb et al., 2015). *BRD4* and related BET proteins regulate the expression of learning and memory-related genes by binding to acetylated histones H4K5/K12ac (Korb et al., 2015; Benito et al., 2017; Sartor et al., 2020). Zhang et al. found in the cell model that inhibiting *BRD4* exacerbates pathological changes in Alzheimer’s disease (AD), leading to Aβ increased sedimentation (Zhang et al., 2022). Button et al. found that selective BET inhibitors can improve cognitive function and alleviate AD symptoms by increasing certain biomarkers (Button et al., 2019).

*PCBP1* belongs to the RNA binding protein in the Poly (C)-binding protein (PCBP) family and contains three conserved KH domains (hnRNP K homology domain). This domain can regulate gene expression at multiple levels by binding to the polycytosine region of RNA (Aasheim et al., 1994; Choi et al., 2009). *PCBP1* can bind to the promoters of multiple genes to regulate gene transcription and can also serve as a splicing factor to regulate selective cleavage of precursor mRNA, thereby affecting the coding and molecular conformation of various proteins (Kim et al., 2005; Jiang et al., 2017; Ji et al., 2018; Wang et al., 2019). Song et al. found that *PCBP1* could enhance or silence the translation process by binding to the internal ribosome entry site regions and 5′ or 3′ untranslated regions of various target mRNA during protein translation (Song et al., 2017; Shi et al., 2018).

*PABPC1* [cytoplasmic poly (A) binding protein 1] is currently the most extensively studied protein in the PABPC family. It participates in many important biological functions such as mRNA metabolism, cell growth and development, and tumor occurrence and development (Kuhn and Wahle, 2004; Goss and Kleiman, 2013). The highly expressed PAIP2 protein in the central nervous system inhibits translation by binding to the PABP domain of *PABPC1* (Berlanga et al., 2006; Uhlen et al., 2015), and is considered a key translation regulatory factor for synaptic plasticity and memory (Khoutorsky et al., 2013). Sarah et al. found that the genetic variation of the *PABPC1* had a higher allele frequency in patients with major depressive disorder (MDD) than in the controls, which may have a potential role in the pathogenesis of MDD (Shyn et al., 2011; Qazi et al., 2022).

*RAG1* (recombination activating gene1) is located on human chromosome 11p12 and has two exons (Li et al., 1997). *RAG1* plays an important role in the immune system’s V (D) J recombination process, and its dysfunction could lead to severe immune function deficiency (Villa et al., 2001; Delmonte et al., 2020). Rosario et al. found extremely rare mutations in *RAG1* in patients with neuropsychiatric syndrome, including frameshift mutations in exons (Trifiletti et al., 2022).

Based on previous evidence of the consistency between gene expression profiles in PBMCs and the brain, this study further explored valuable DEGs in leukocytes for SCZ, and identified three functional genes *BRD4*, *PABPC1*, and *RAG1* with various interpretability in terms of possible pathogenic mechanisms underline SCZ. Nevertheless, it is necessary to refine the DEGs from GO enrichment analysis, KEGG pathway enrichment analysis, and weighted gene co-expression network analysis for further research and validation. The potential pathogenic effect of other DEGs on SCZ remains to be clarified in the future. Since we identified several key genes from peripheral leukocytes, the implemental value of DEGs of five gene modules warrants further evaluation in the diagnosis and prognosis of SCZ.

The RNA sequencing in this study was conducted on peripheral blood samples; nonetheless, the DEGs identified in this study continue to overlap with GWAS and the *postmortem* brain tissue sequencing investigations (Supplementary Table S6; Supplementary Figure S2). And the DEGs also overlap with the risk genes for SCZ found in relevant databases (Supplementary Figure S3). Some genes, including *RERE*, have been shown in earlier investigations to be substantially related with SCZ. Nevertheless, the remaining DEGs should be verified in a larger population because to the sequencing sample size constraint.

In conclusion, our findings suggest that DEGs in leukocytes exhibit a significant discriminative value for SCZ, underlining the biological functions in key modules and related enrichment pathways help to elucidate the molecular pathogenesis. The potential of the key genes as biomarkers for diagnosis and prognosis of SCZ warrants further research in the future.

## Data Availability

Original datasets are available in a publicly accessible repository: Gene Expression Omnibus data base (GEO). The original contributions presented in the study are publicly available. This data can be found here: GSE263180.
