# Supplementary material for: Identification of differentially expressed genes of blood leukocytes for Schizophrenia
Source: Front Genet. 2024 Jun 26;15:1398240. doi: 10.3389/fgene.2024.1398240 (PMC11233772; doi:10.3389/fgene.2024.1398240)
Supplement: Supplementary file 2 [file Table1.DOCX]

**Supplementary Information**

**Identification of Differentially Expressed Genes of Blood Leukocytes for Schizophrenia**

Feifan Wang^1†^, Yao Fan^2†^, Yinghui Li^3^, Yuan Zhou^1^, Xin Wang^4^, Mengya Zhu^4^, Xuefei Chen^4^, Yong Xue^4^*, Chong Shen^1^*

^1^ Department of Epidemiology, School of Public Health, Nanjing Medical University, Nanjing, China

^2^ Department of Clinical Epidemiology, Jiangsu Province Geriatric Institute, Geriatric Hospital of Nanjing Medical University, Nanjing, China

^3^ Department of Medical Psychology, Huai’an Third Hospital, Huai'an, China

^4^ Department of Medical Laboratory, Huai’an Third Hospital, Huai'an, China

^†^ These authors contributed equally to this work and share the first authorship.

* Correspondence: Chong Shen, sc100@126.com; Yong Xue, xueyong3@126.com

**Table S1 Demographic characteristics of SCZ cases and controls.**

|  | SCZ (n=9) | HC (n=20) |
| --- | --- | --- |
| Age | 48.50 (45.25-51.00) | 66 (65.75-68.00) |
| Sex (M/F) | 3/6 | 10/10 |

Note: SCZ, schizophrenia; HC, health controls; M, male; F, female.

**Table S2 Functional and pathway enrichment of DEGs in schizophrenia**

**GO_BP (Top10)**

| ID | Description | *P*-value | Count |  |
| --- | --- | --- | --- | --- |
| GO:0060326 | cell chemotaxis | 1.65E-06 | 37 |  |
| GO:0098609 | cell-cell adhesion | 2.04E-05 | 74 |  |
| GO:0015988 | energy coupled proton transmembrane transport,  against electrochemical gradient | 3.28E-05 | 4 |  |
| GO:0015990 | electron transport coupled proton transport | 3.28E-05 | 4 |  |
| GO:0006935 | chemotaxis | 3.44E-05 | 58 |  |
| GO:0042330 | taxis | 3.76E-05 | 58 |  |
| GO:2000502 | negative regulation of natural killer cell chemotaxis | 1.34E-04 | 3 |  |
| GO:1900153 | positive regulation of nuclear-transcribed mRNA catabolic process, deadenylation-dependent decay | 1.94E-04 | 6 |  |
|  |  |  |  |  |
| GO:0032496 | response to lipopolysaccharide | 2.41E-04 | 33 |  |
| GO:0055083 | monovalent inorganic anion homeostasis | 2.52E-04 | 7 |  |

**GO_CC (Top10)**

| ID | Description | *P*-value | Count |
| --- | --- | --- | --- |
| GO:0009986 | cell surface | 3.06E-05 | 76 |
| GO:0070161 | anchoring junction | 6.48E-04 | 65 |
| GO:0031012 | extracellular matrix | 1.96E-03 | 46 |
| GO:0005911 | cell-cell junction | 2.08E-03 | 41 |
| GO:0070469 | respirasome | 2.18E-03 | 13 |
| GO:0098803 | respiratory chain complex | 4.44E-03 | 11 |
| GO:0062023 | collagen-containing extracellular matrix | 5.31E-03 | 35 |
| GO:0032591 | dendritic spine membrane | 6.23E-03 | 4 |
| GO:0005746 | mitochondrial respirasome | 6.30E-03 | 11 |
| GO:0097450 | astrocyte end-foot | 6.43E-03 | 3 |

**GO_MF (Top10)**

| ID | Description | *P*-value | Count |
| --- | --- | --- | --- |
| GO:0035925 | mRNA 3'-UTR AU-rich region binding | 3.34E-04 | 7 |
| GO:0017091 | AU-rich element binding | 4.30E-04 | 7 |
| GO:0035662 | Toll-like receptor 4 binding | 5.90E-04 | 3 |
| GO:0047115 | trans-1,2-dihydrobenzene-1,2-diol dehydrogenase activity | 5.90E-04 | 3 |
| GO:0008236 | serine-type peptidase activity | 1.10E-03 | 21 |
| GO:0016655 | oxidoreductase activity, acting on NAD(P)H, quinone or | 1.40E-03 | 10 |
|  | similar compound as acceptor |  |  |
| GO:0017171 | serine hydrolase activity | 1.44E-03 | 21 |
| GO:0004252 | serine-type endopeptidase activity | 1.83E-03 | 19 |
| GO:0005501 | retinoid binding | 2.65E-03 | 7 |
| GO:0023024 | MHC class I protein complex binding | 2.72E-03 | 3 |

**KEGG (Top 10)**

| ID | Description | *P*-value | Count |
| --- | --- | --- | --- |
| hsa05012 | Parkinson's disease | 8.24E-04 | 17 |
| hsa00190 | Oxidative phosphorylation | 2.60E-03 | 16 |
| hsa04650 | Natural killer cell mediated cytotoxicity | 3.52E-03 | 16 |
| hsa00980 | Metabolism of xenobiotics by cytochrome P450 | 5.64E-03 | 10 |
| hsa04742 | Taste transduction | 7.55E-03 | 8 |
| hsa05332 | Graft-versus-host disease | 8.97E-03 | 7 |
| hsa05143 | African trypanosomiasis | 1.19E-02 | 6 |
| hsa04380 | Osteoclast differentiation | 2.56E-02 | 13 |
| hsa05215 | Prostate cancer | 2.57E-02 | 10 |
| hsa00910 | Nitrogen metabolism | 3.64E-02 | 4 |

Notes: GO, gene ontology; BP, biological process; CC, cellular component; MF; molecular function; KEGG, Kyoto Encyclopedia of Genes and Genomes pathway analysis.

**Table S3 Numbers of genes distributed in modules**

| module | genes |
| --- | --- |
| greenyellow | 78 |
| grey | 937 |
| yellow | 667 |
| green | 327 |
| salmon | 43 |
| blue | 1590 |
| cyan | 39 |
| turquoise | 4823 |
| brown | 676 |
| pink | 157 |
| tan | 56 |
| purple | 97 |
| black | 173 |
| red | 184 |
| magenta | 117 |
| midnightblue | 36 |

**Table S4 Key genes in key modules**

| pink (UP) | | purple (UP) | tan (UP) | turquoise (DOWN) | | black (DOWN) |
| --- | --- | --- | --- | --- | --- | --- |
| LMOD2 | DPM3 | TULP2 | CCIN（Up） | ARHGEF28 | UBE2T | SMAD9 |
| IFNL1 | PABPC1 | FABP6 | TAS1R1 | FGF17 | ARHGEF19 | JAM2 |
| LPPR3 | TPP1 | AGAP2-AS1 | GPR3 | ARVCF | CASP16 | RGS17 |
| MUC1 | TGFB1 | MYADM | PDLIM4 | MYO6 | RAG1 | FAM150B |
| DNAJB13 | RPL41 | SLC22A1 | TMC3 | DHCR24 | C3orf18 | FILIP1 |
| RPS21 | HPRT1 | AL590560.1 | AC132872.2 | B3GAT1 | METRN | AGRP |
| POU2AF1 | EIF5A | CRABP2 | AL590714.1 | PTPRM | PYHIN1 | VASH2 |
| VAMP2 | HNRNPH3 | CBFA2T3 | KLLN | JAKMIP2 | CRYGS | ALDH1L2 |
| EIF1B | HNRNPD | KDM6B | RLBP1 | GLB1L2 | DISP2 | SLC35G2 |
| RCCD1 | GATAD2B | DOK3 | TREML4 | TTC16 | USP28 |  |
| SMDT1 | BRD4 | MBD6 | GSTO2 | GZMH | ZNF234 |  |
| RPS28 | POM121C | FBXO24 | APOA2 | FCRL6 | ZNF30 |  |
| ATN1 | PITPNB | CCDC36 | CSNK1D | EOMES | SPTBN5 |  |
| GLTSCR1 | CTDNEP1 | BASP1 | CCDC96 | FAM179A | LRRC46 |  |
| USB1 | VPS26A | LY96 | RABGEF1 | PIF1 | MIB2 |  |
| ZNF385A |  | RFX2 | U2AF1 | ADAMTS4 | ZNF584 |  |
| FUS |  | APOBR | KIRREL3 | GPR56 | AC010536.1 |  |
| RPL27A |  | CREB5 | DCST2 | C1orf21 | ZNF232 |  |
| NAP1L5 |  | KCND1 | IL27 | CUZD1 | TTC22 |  |
| RPL28 |  | CSF2RA | MIDN | PRF1 | CCDC78 |  |
| RPL27 |  | PAM16 | TMEM184A | FGFBP2 | ZNF324B |  |
| PCBP2 |  | F5 | IPO4 | SPAG8 | MFSD3 |  |
| SP2 |  | RAB7A | CCNK | CEP78 | PYCRL |  |
| STX16 |  | TCF7L2 | KIAA0895 | ERBB2 | ZNF546 |  |
| F8A1 |  | PANX2 | EIF1 | GK5 | ZNF583 |  |
| PCBP1 |  | NFIC | AC087239.1 | CACNA2D2 | JMJD4 |  |
| ANKDD1A |  | AKIRIN2 | SF1 | ZMYND10 | ZNF225 |  |
| HNRNPA3 |  | KLF6 | C8orf58 | SGSM1 | TSPAN32 |  |
| RNF26 |  | UBE2R2 | MAP1LC3B | LEPREL4 | C9orf96 |  |
| POLR2A |  | PRKCD |  | PDGFD | PAFAH2 |  |
| GNG5 |  | BRI3 |  | CDHR3 | N6AMT1 |  |
|  |  |  |  | ZNF600 | SLC26A1 |  |
|  |  |  |  | CARNS1 | MRM1 |  |
|  |  |  |  | SLC2A12 | TONSL |  |
|  |  |  |  | SOX13 | LTB4R2 |  |
|  |  |  |  | TTC38 | GFI1 |  |
|  |  |  |  | LLGL2 | FABP3 |  |
|  |  |  |  | SLC34A3 | MMACHC |  |
|  |  |  |  | OSBPL5 | MLC1 |  |
|  |  |  |  | TLR3 | PLEKHF1 |  |
|  |  |  |  | TBX21 |  |  |

**Table S5 Functional and pathway enrichment of 10 genes**

**GO_BP**

| ID | Description | *P*-value | Count |
| --- | --- | --- | --- |
| GO:0002181 | cytoplasmic translation | 8.15093E-06 | 4 |
| GO:0006412 | translation | 1.12E-04 | 4 |
| GO:0039694 | viral RNA genome replication | 0.004157038 | 2 |
| GO:0051252 | regulation of RNA metabolic process | 0.008757947 | 2 |
| GO:0048255 | mRNA stabilization | 0.016080109 | 2 |

**GO_CC**

| ID | Description | *P*-value | Count |
| --- | --- | --- | --- |
| GO:0005840 | ribosome | 4.76E-05 | 4 |
| GO:1990904 | ribonucleoprotein complex | 6.28E-05 | 4 |
| GO:0005654 | nucleoplasm | 2.87E-04 | 8 |
| GO:0022626 | cytosolic ribosome | 7.08E-04 | 3 |
| GO:0098556 | cytoplasmic side of rough endoplasmic reticulum membrane | 0.002177805 | 2 |
| GO:0005737 | cytoplasm | 0.00273698 | 8 |
| GO:0070062 | extracellular exosome | 0.011110092 | 5 |
| GO:0015935 | small ribosomal subunit | 0.012141505 | 2 |
| GO:0042788 | polysomal ribosome | 0.013865265 | 2 |
| GO:0005925 | focal adhesion | 0.014391435 | 3 |

**GO_MF**

| ID | Description | *P*-value | Count |
| --- | --- | --- | --- |
| GO:0003723 | RNA binding | 5.41E-07 | 8 |
| GO:0003735 | structural constituent of ribosome | 7.95E-05 | 4 |
| GO:0003729 | mRNA binding | 0.006090219 | 3 |
| GO:0005515 | protein binding | 0.026216706 | 10 |

**KEGG**

| ID | Description | *P*-value | Count |
| --- | --- | --- | --- |
| hsa03010 | Ribosome | 3.69E-04 | 4 |
| hsa05171 | Coronavirus disease - COVID-19 | 9.67E-04 | 4 |
| hsa04216 | Ferroptosis | 0.03733627 | 2 |

Notes: GO, gene ontology; BP, biological process; CC, cellular component; MF; molecular function; KEGG, Kyoto Encyclopedia of Genes and Genomes pathway analysis.

**Table S6 DEGs overlapped with published studies**

| DEGs overlapped with published studies | PMID |
| --- | --- |
| PITPNB, ZNF385A, VPS26A, ACTR2, HNRNPH3, KIFC2, LCMT2, PGRMC2, JMJD4, PPTC7, SLC38A5, PTP4A1, FEM1C, METRN, PAPLN, PYCRL, POLR3G, BFSP1, GNG7, S100A1, ACOT4, RTN4R, BCL6, ARHGEF28, SCN1B, PPP1R1A, CA4, ALPL, CHRM4, LYNX1, C21orf62, ZNF835, RNF150, KNDC1, C1orf61, CPLX1, SLC25A10, IGF1, KREMEN2, FGFR3, CLEC14A | 27668389 |
| NFIC, LMOD2, TBX21, KLLN, FASLG, MMACHC, P4HA2, SEC14L3, ULBP3, USHBP1, SPC25, IDO1, ASB15, CCDC168, MAB21L3, ALPK2, HOXB7, HOXA10, TNFSF15, PLEKHS1, LAD1, LDB3, BEND6, BOLA2B, ZBTB7C, RHOXF2, NLRP14, ZSCAN23, GFPT2 | 37142572 |
| S100A10, MTRNR2L9, RBPMS2, IFITM2, GK5, ARHGEF19, C1R, COL4A1 | 34440415 |
| RERE, KLF6, PAK6, SGCD, ZNF835 | 35396580 |

**Table S7 The correlation between genes and age** **in two pathways**

| pathway | DEGs | r | P | Up or Down in SCZ |
| --- | --- | --- | --- | --- |
| Oxidative phosphorylation | ATP4A | 0.458 | **0.012** | down |
|  | ATP6V0C | -0.101 | 0.601 | up |
|  | MT-ATP6 | -0.394 | **0.034** | up |
|  | MT-ATP8 | -0.441 | **0.017** | up |
|  | MT-CO1 | 0.176 | 0.362 | up |
|  | MT-CYB | -0.443 | **0.016** | up |
|  | MT-ND5 | -0.5 | **0.006** | up |
|  | MT-ND6 | -0.265 | 0.165 | up |
|  | NDUFA4L2 | 0.383 | **0.041** | down |
|  | NDUFB3 | 0.296 | 0.119 | up |
| Cytotoxicity | FASLG | 0.708 | **＜0.001** | down |
|  | GZMB | 0.573 | **0.001** | down |
|  | KIR2DL1 | 0.382 | **0.041** | down |
|  | KLRK1 | 0.76 | **＜0.001** | down |
|  | PPP3R1 | 0.263 | 0.169 | up |
|  | PRF1 | 0.599 | **0.001** | down |
|  | PRKCG | -0.546 | **0.002** | up |
|  | SH2D1B | 0.535 | **0.003** | down |
|  | ULBP2 | 0.315 | 0.096 | down |
|  | ZAP70 | 0.577 | **0.001** | down |

**Figure S1 Research flowchart**


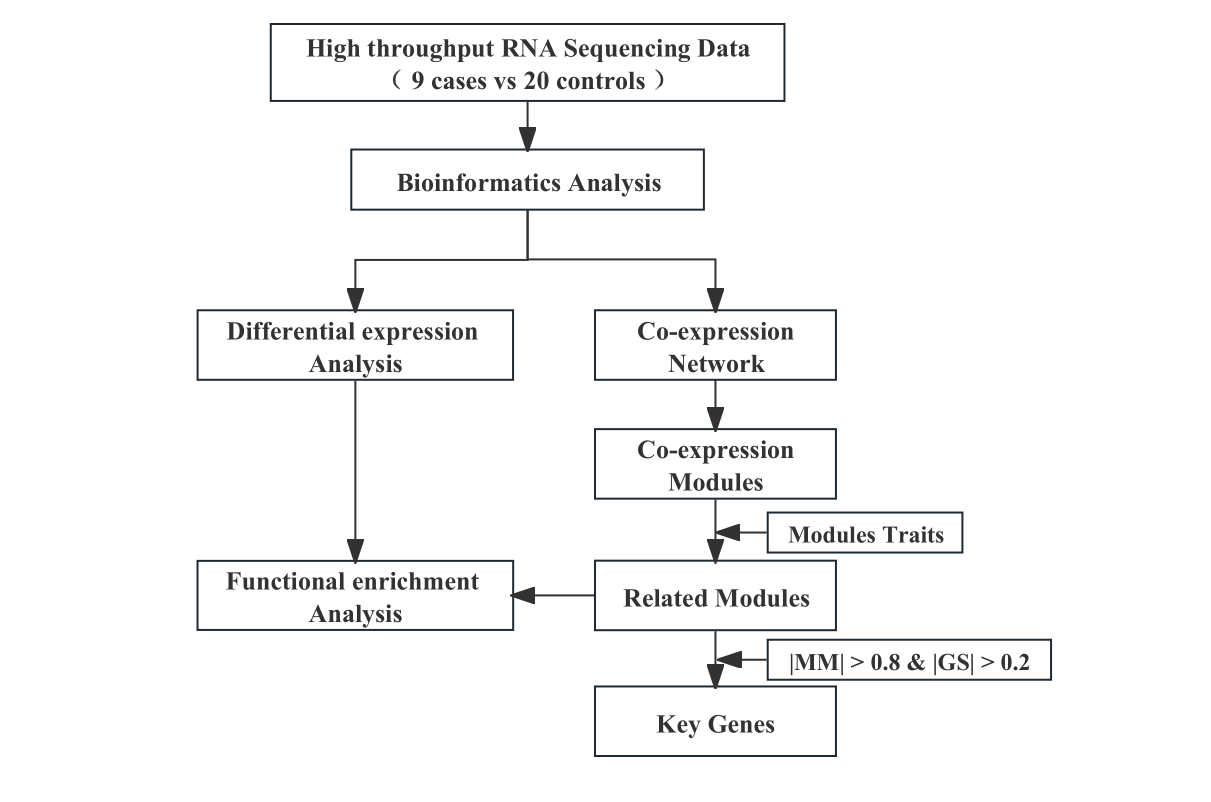


**Figure S2 Venn diagrams of DEGs overlapped with published studies**


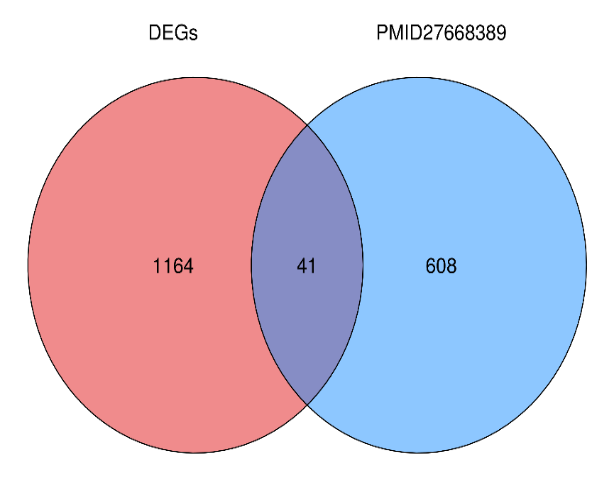

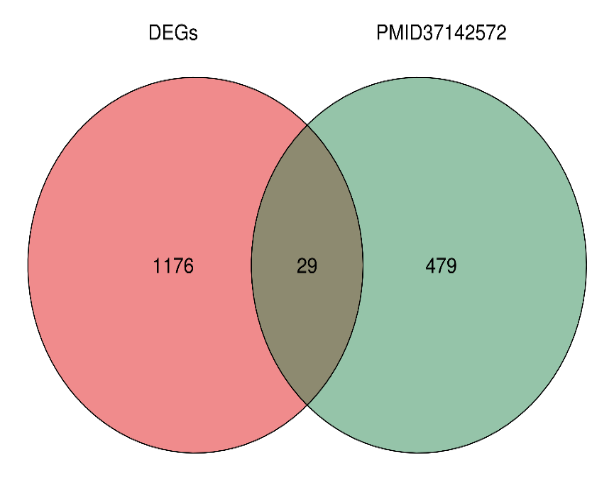


**A B**


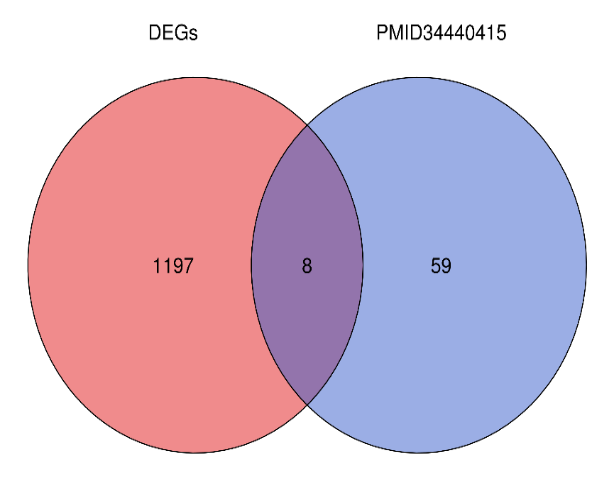

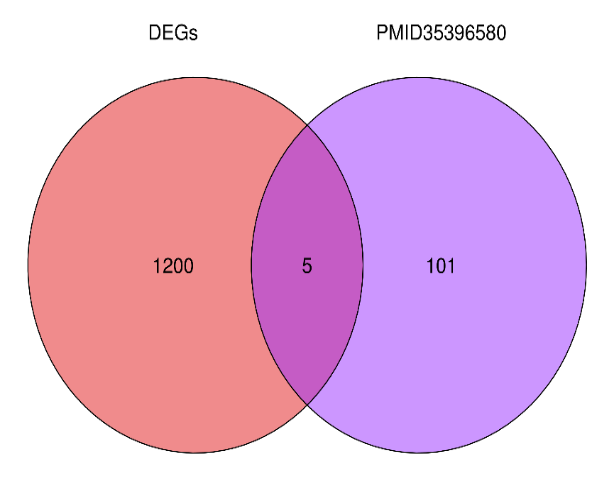


**C D**

**Figure S3 Venn diagrams of DEGs overlapped with SCZ risk gene sets**


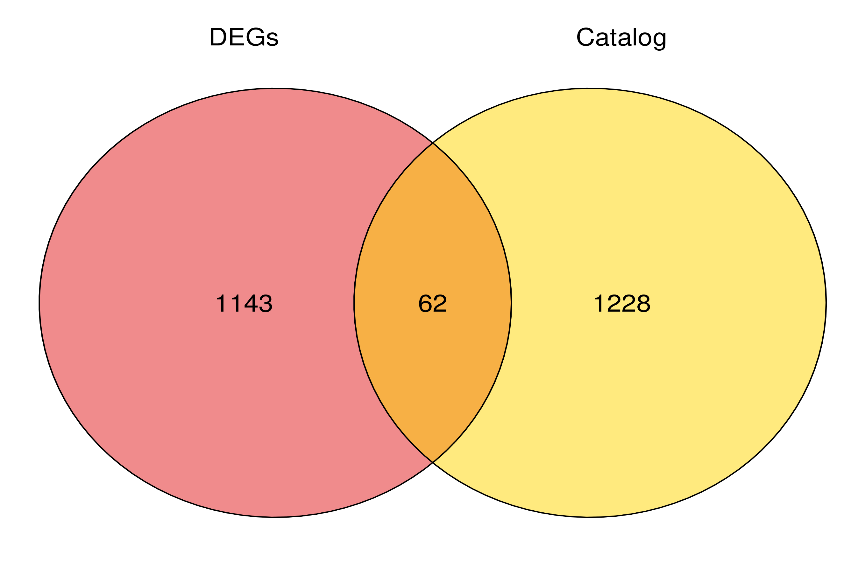


**A**


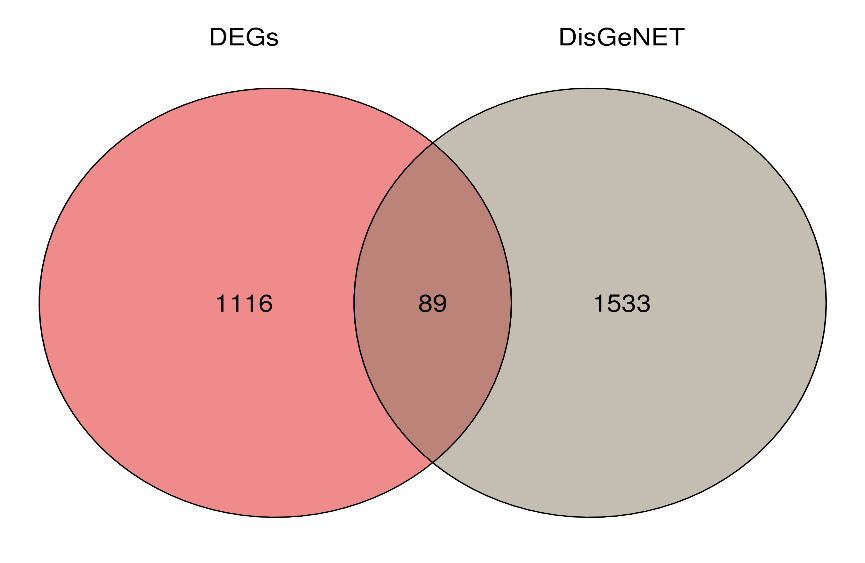


**B**


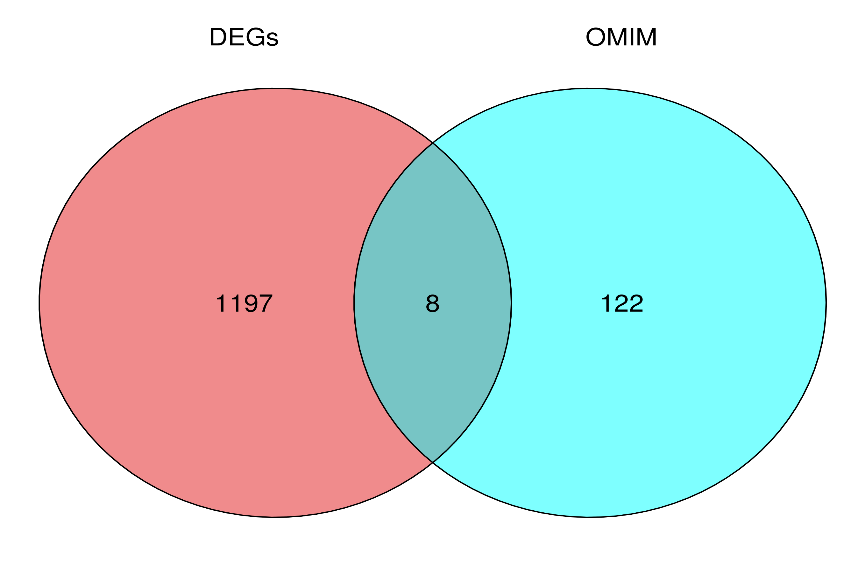


**C**
