# Supplementary figures and images for: Identification of differentially expressed genes of blood leukocytes for Schizophrenia
Source: Front Genet. 2024 Jun 26;15:1398240. doi: 10.3389/fgene.2024.1398240 (PMC11233772; doi:10.3389/fgene.2024.1398240)

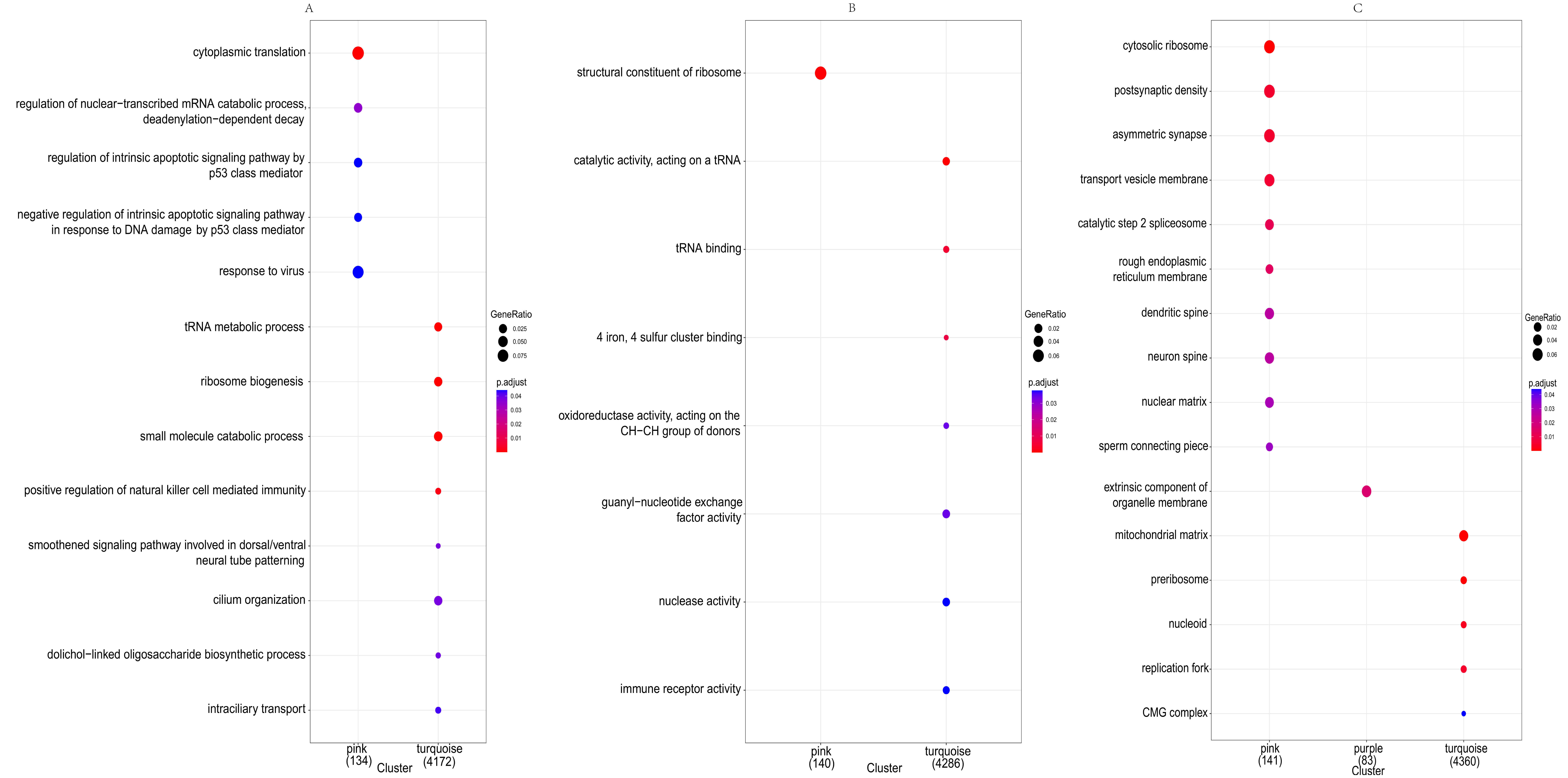

Supplement: Supplementary file 3 [file Image5.tiff]
